# Development and clinical application of an Intelligent Operating Room Scheduling System

**DOI:** 10.3389/fmed.2026.1848933

**Published:** 2026-07-21

**Authors:** Hong Liang, Fengping Zhang, Liwei Huang, Jie Zhao, Sijia Wu, Ruiping Na, Jianxia Ma, Lihong Hu, Yan Sun

**Affiliations:** 1 Department of Anesthesia and Perioperative Medicine, General Hospital of Ningxia Medical University, Yinchuan, China; 2 Department of Anesthesia and Perioperative Medicine, Cardiovascular and Cerebrovascular Disease Hospital, General Hospital of Ningxia Medical University, Yinchuan, China; 3 Department of Information Center, General Hospital of Ningxia Medical University, Yinchuan, China; 4 Department of Geriatrics, General Hospital of Ningxia Medical University, Yinchuan, China

**Keywords:** digital health, healthcare management, hospital information system, nurse scheduling, operating room scheduling, resource allocation

## Abstract

**Objective:**

To address inefficiencies of traditional operating room scheduling, including excessive time, low subspecialty matching accuracy, and inequitable performance compensation, we developed an Intelligent Operating Room Scheduling System (IORSS) based on the hospital information system (HIS) and evaluated its clinical effects.

**Methods:**

System requirements were identified via literature review, interviews, and expert panels. Using agile development, we built an HIS-based IORSS with three modules: rapid room assignment, nurse subspecialty matching, and automated attendance management. A quasi-experimental pre-/post-implementation study compared control (Jan–Jun 2025, manual scheduling) and experimental (Jul–Dec 2025, IORSS-assisted) groups. Outcomes were scheduling time, matching accuracy, and satisfaction with performance distribution fairness.

**Results:**

Scheduling time decreased from 7.0 ± 1.2 to 2.0 ± 0.5 h (*P* < 0.01); subspecialty matching accuracy improved from 78.6% to 94.3% (*P* < 0.01); satisfaction with performance fairness increased from 61.5% to 88.3% (*P* < 0.05).

**Conclusion:**

The HIS-based IORSS significantly reduces scheduling time, improves nurse-to-surgical demand matching, and enhances satisfaction with performance distribution fairness. It effectively boosts OR resource utilization and is ready for broader adoption.

## Introduction

The operating room (OR) is the hospital’s surgical hub; its efficiency directly affects institutional performance and patient experience. OR operations may contribute 40%–70% of hospital revenue (1). Surgical scheduling critically determines resource utilization, turnover, overtime, and patient waiting. However, most hospitals still perform manual scheduling by head nurses, which is time consuming, subjective, and prone to imbalanced workloads and poor subspecialty matching. At our institution, manual scheduling took approximately 7 h per day based on preliminary observation.

Our institution previously used a hybrid block/open scheduling system. In block scheduling, specific ORs were reserved for certain surgical teams on fixed weekdays; in open scheduling, remaining time slots were filled on a first-come, first-served basis. The main challenges included: (1) frequent overbooking leading to prolonged waiting times, (2) mismatch between nurse subspecialty skills and surgical demands, and (3) lack of transparency in nurse assignment, which caused dissatisfaction with performance fairness. Anesthesia personnel were not included in this study because they operate under a separate scheduling system with dedicated staffing pools and different shift structures (e.g., 24-h on-call rotations) that are not integrated with the HIS. Including them would have required a separate module beyond the scope of this initial implementation. Consequently, our findings reflect improvements in nursing and OR assignment efficiency but do not capture the full spectrum of OR team coordination, which would require integration with anesthesia scheduling systems in future work.

Recent advances in operations research and AI have improved surgical scheduling. Heuristic algorithms and integer programming remain core themes (2, 3). Reinforcement learning (RL) and hybrid frameworks have shown reductions in patient waiting time and increased OR utilization (4, 5). Machine learning (ML) models predict surgical duration more accurately than traditional methods (6). Commercial platforms (e.g., Qventus, LeanTaaS) have demonstrated real-world benefits (7, 8). Unlike algorithmic studies that focus on theoretical optimization, our system addresses practical implementation challenges by integrating nursing subspecialty matching and performance linkage into a rule-based HIS platform.

Despite these advances, three gaps remain: (1) Most studies focus on algorithmic optimization, not integration with nursing management, particularly subspecialty matching and performance linkage. (2) Real-world implementation reports are scarce; systems often poorly adapt to hospital workflows. (3) To our knowledge, no mature rule-based scheduling system has been implemented in our institution or region, despite the region being designated as the first “Internet + Healthcare” demonstration zone in China.

From a Digital Public Health perspective, OR scheduling efficiency directly impacts health system performance by influencing surgical throughput, workforce utilization, and patient access to timely surgical care. Improved scheduling can reduce surgical waiting times and potentially mitigate health disparities related to delayed access to surgery. To address these gaps, we developed an HIS-based IORSS with three modules: rapid OR assignment, subspecialty-based nurse scheduling, and automated attendance-performance management. We evaluated its operational performance in a pre-/post-implementation study.

## Materials and methods

### Study design

**FIGURE 1:**
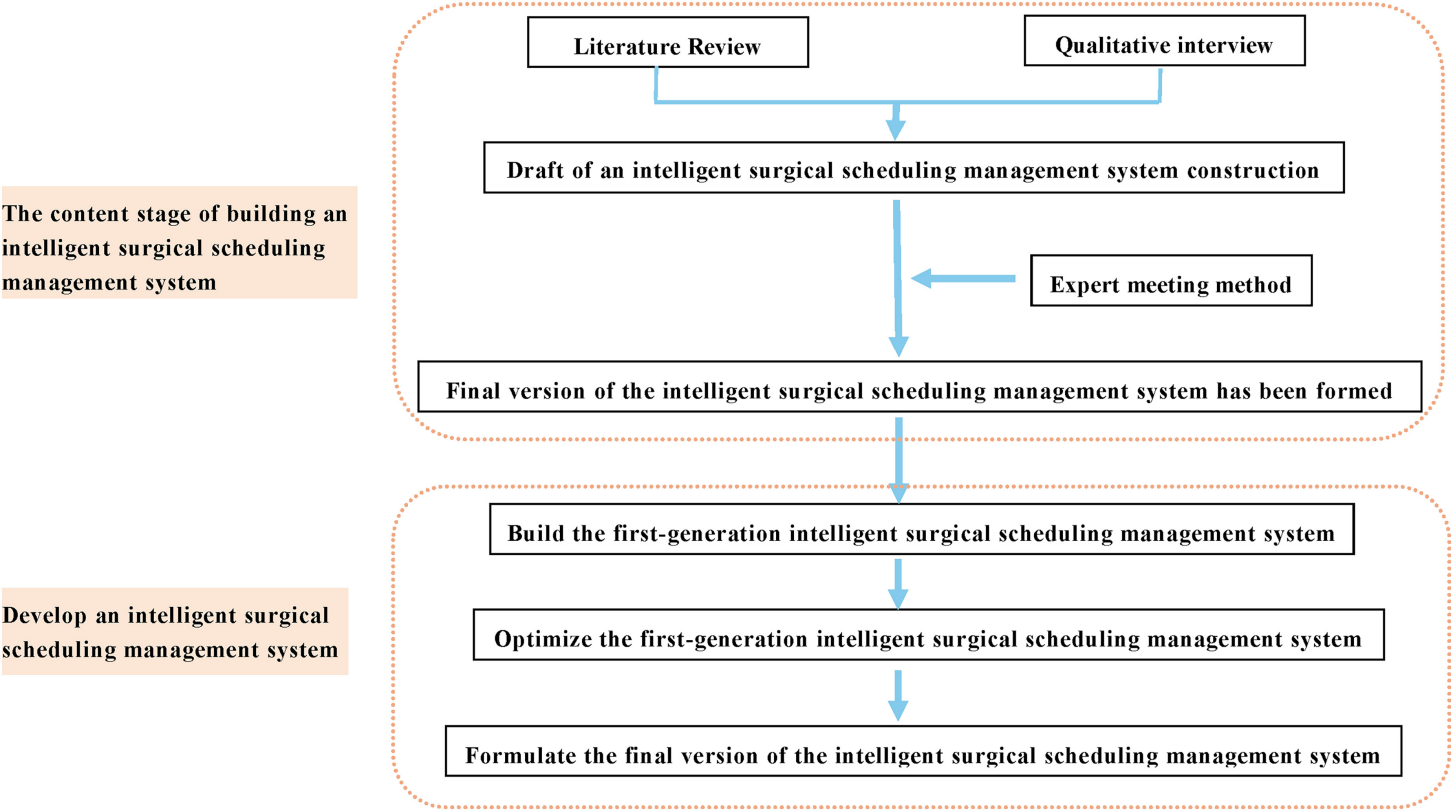
Research design technical roadmap.

This study was conducted in two phases. The first phase involved the design and development of the Intelligent Operating Room Scheduling System (IORSS). System functional requirements were identified through literature review, clinical needs analysis, and expert panel meetings, and the system was constructed using agile software development methods. The second phase was a quasi-experimental study with a pre-/post-implementation design to evaluate the application effects of the system in operating room nursing scheduling. The research design and technical roadmap are illustrated in Figure 1. The reporting of this quality improvement initiative follows the SQUIRE 2.0 (Standards for Quality Improvement Reporting Excellence) guideline. The study protocol was reviewed and deemed exempt from formal ethical approval by the Ethics Committee of the General Hospital of Ningxia Medical University (filing number: KYLL-2025-0791), and the study adhered strictly to the principles of the Declaration of Helsinki.

### Design and development

#### Requirements analysis

We systematically searched CNKI/PubMed (2006–2024) for scheduling modules and rules. Semi-structured interviews were conducted with surgeons (*n* = 6), OR nurses (*n* = 10), and managers (*n* = 4). Six experts (≥15 years’ experience) convened to finalize three core modules and scheduling rules. Based on the literature review, interviews, and expert panel consensus, we identified the following functional requirements: (1) automated OR assignment based on surgeon-team-department hierarchy, (2) nurse scheduling based on 12 subspecialty groups with a general pool for flexible coverage, and (3) automated attendance tracking linked to performance scoring. The agile development process incorporated iterative feedback from end-users during each sprint. The 2-weeks pilot testing phase further refined the user interface and rule parameters based on real-time user feedback.

#### System development

**FIGURE 2:**
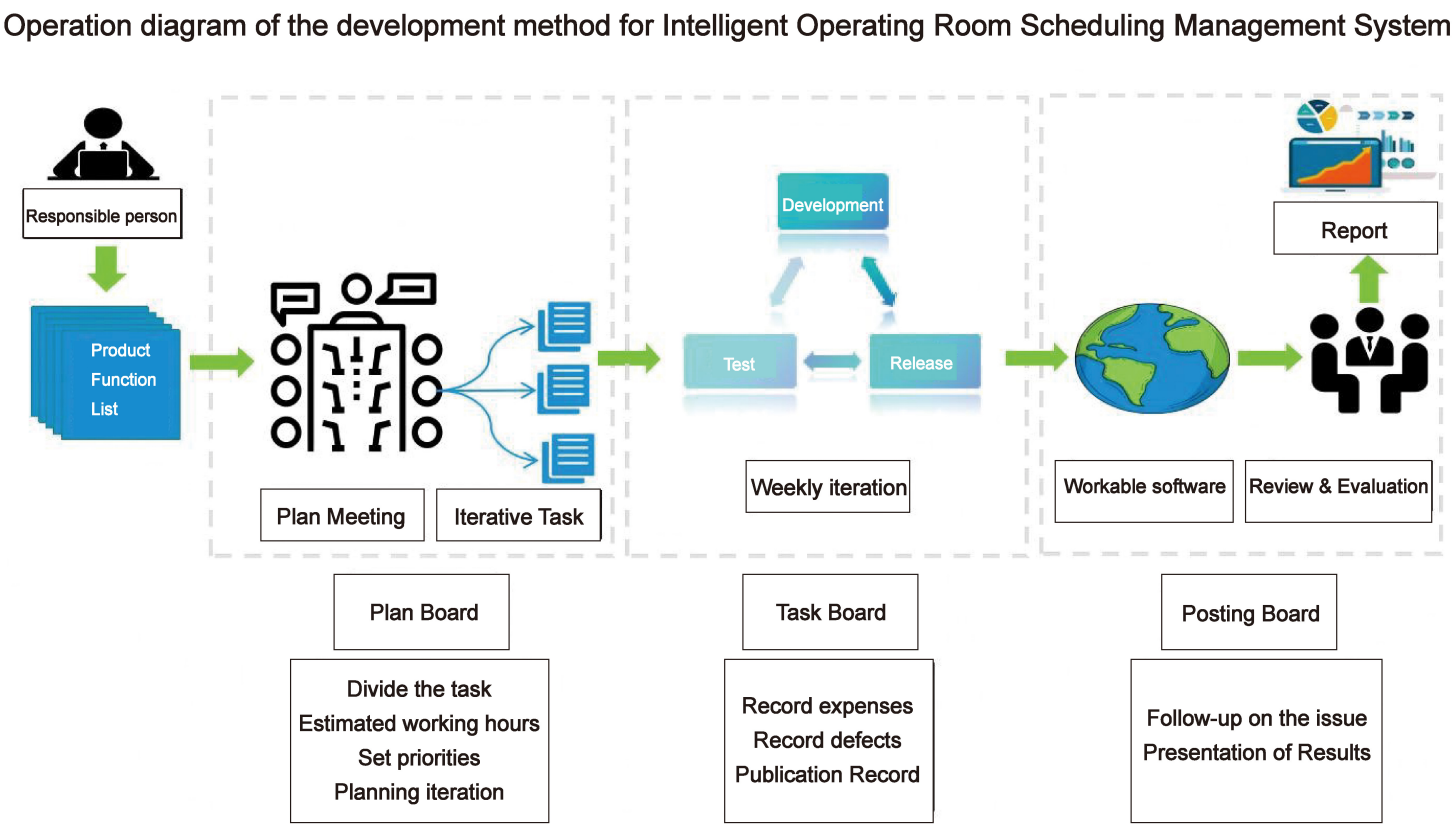
Software development flowchart.

The system extends the existing HIS using a three-tier architecture (Java back-end, Oracle database). Agile methods enabled rapid iteration (the software development process is shown in Figure 2). The front-end is embedded in the HIS portal. It is important to note that the current version of IORSS operates on rule-based logic (priority matching: individual surgeon → team leader → department) and does not employ machine learning or artificial intelligence models. In this context, “intelligent” refers to automated rule-based decision support that reduces manual effort and improves scheduling transparency, rather than adaptive or predictive AI capabilities.

### System functional modules

#### Rapid operating room assignment module

Based on the hospital’s operating day scheduling principles, a weekly correspondence was established between operating rooms and individual surgeons, surgical teams, or clinical departments. The assignment rules were as follows: priority was given to matching the individual surgeon, followed by the surgical team leader, and finally the surgical department. Special surgeries (e.g., robotic surgery, day surgery) were flagged by the surgeon at the time of request, and the system automatically assigned them to the appropriate operating rooms. Scheduling personnel could click “one-click operating room assignment” to complete automated allocation, with manual fine-tuning supported. The rule-based assignment takes into account the following factors: (1) a weekly pre-defined mapping table linking individual surgeons, surgical teams, or departments to specific operating rooms; (2) special surgery flags (e.g., robotic surgery, day surgery) submitted by the surgeon at the time of request; and (3) daily operating room capacity limits. No machine learning was applied to historical schedule records.

#### Subspecialty-based nurse scheduling module

A total of 130 operating room nurses were assigned to 12 subspecialty groups (e.g., orthopedics, general surgery, neurosurgery). Nurses with lower seniority or those not yet assigned to a fixed subspecialty were placed into a general pool. The system generated a monthly duty roster for the department, including administrative shifts, on-call shifts, post-night shifts, standby shifts, and leave. Temporary absences were updated in real time by the head nurse; the system automatically excluded unavailable personnel for the day and generated a list of available nurses. A match was considered “successful” if the assigned nurse belonged to the subspecialty group corresponding to the surgical department (e.g., orthopedics nurse for orthopedic surgery). Cross-group assignment was allowed only when the primary subspecialty group had no available nurses; such assignments were recorded as “mismatched” in the accuracy calculation. Additionally, nurses in the general pool were not considered subspecialty-matched for any specialty surgery. For high-risk patients (e.g., age ≥ 70 years), the system automatically flagged them with a color code to prompt scheduling personnel to assign experienced nurses. This color-coding function was implemented as part of the system but was not formally evaluated as a separate outcome in this study.

#### Nurse attendance management module

Daily scheduling data were linked to attendance records, automatically generating monthly attendance summaries that were synchronized with the performance distribution system, thereby achieving integrated management of scheduling, attendance, and performance. The performance score for each nurse was calculated based on attendance (30%), workload (number of surgical cases participated, 40%), and subspecialty matching compliance (30%). These weights were determined by the expert panel (*n* = 6) based on institutional priorities for nurse performance evaluation: workload was given the highest weight (40%) to incentivize clinical participation, while attendance (30%) and subspecialty matching compliance (30%) were weighted equally to balance reliability and quality of care. The system generated a monthly performance report automatically; no manual adjustment was allowed unless approved by the department head.

#### System testing and deployment

After development was completed, a 2-weeks clinical pilot test was conducted in the operating room. Iterative fixes were made in response to user feedback, and the system was officially released for hospital-wide implementation following successful testing.

### Clinical evaluation methods

#### Study participants

Convenience sampling. Control group: Jan–Jun 2025 (manual scheduling). Experimental group: Jul–Dec 2025 (IORSS-assisted). No significant differences in active ORs, nurses on duty, or proportion in subspecialty groups between periods (*P* > 0.05).

#### Sample size calculation

Assuming a two-sided α = 0.05, power = 0.80, and a 20% absolute improvement in satisfaction (from ~60% to ~80%), a minimum of 62 nurses per group was required. The baseline satisfaction of ~60% was estimated from a preliminary survey of 30 nurses conducted in December 2024 (data not shown), and a 20% improvement was considered clinically meaningful based on literature. Satisfaction with performance distribution fairness was one of three co-primary outcomes (alongside scheduling time and matching accuracy).

#### Intervention

The surgical request process was identical in both groups: surgeons submitted surgical requests through the HIS before 10:30 AM daily; after approval by the department director, the requests were automatically pushed to the operating room scheduling interface.

Control group: The head nurse or scheduling specialist manually assigned operating rooms and nursing staff to each surgical case based on the requests displayed on the interface. This process took approximately 7 h per day.

Experimental group: The scheduling personnel clicked the “one-click operating room assignment” button, and the system automatically allocated operating rooms according to predefined rules. Subsequently, they clicked the “one-click nurse assignment” button, and the system automatically assigned nursing staff based on subspecialty matching rules, with high-risk patients automatically flagged. Scheduling personnel could make manual adjustments as needed for special requirements. At the end of each month, clicking the “attendance summary” button automatically generated the monthly attendance summary for nursing staff.

#### Outcome measures

Scheduling time: The total time (in hours) from when scheduling personnel began processing surgical requests until all operating rooms and nursing staff assignments were completed. Scheduling time was measured by an independent research assistant using the system’s timestamp logs. The start time was recorded when the scheduling personnel began processing surgical requests, and the end time was recorded when all assignments were confirmed. The unit of analysis for this outcome was scheduling days (182 days in the control group and 184 days in the experimental group).

Subspecialty matching accuracy: The proportion (%) of surgical cases in which the nurse’s assigned subspecialty matched the surgical department’s specialty requirements, calculated as (number of matched cases/total surgical cases) × 100%. The unit of analysis for this outcome was individual surgical cases.

Satisfaction with performance distribution fairness: A self-developed satisfaction questionnaire was used. The content validity was evaluated by five experts, with a content validity index (CVI) of 0.92, and the internal consistency reliability (Cronbach’s α) was 0.87. The questionnaire assessed the proportion (%) of nursing staff satisfied with the fairness of performance distribution. The survey was administered anonymously at the end of each phase. The questionnaire was distributed to all 130 nurses on duty in each phase; 126 valid responses were received in the control group (response rate 96.9%) and 128 in the experimental group (response rate 98.5%). The same nurses were surveyed in both phases to ensure comparability, although individual responses were anonymized. The unit of analysis for this outcome was individual nurse respondents.

### Statistical analysis

Statistical analyses were performed using SPSS version 27.0 (IBM Corp., Armonk, NY, USA). Continuous data were first tested for normality using the Shapiro–Wilk test. Data that followed a normal distribution were presented as mean ± standard deviation ($\bar{\text{x}}$ ± s), and between-group comparisons were conducted using the independent-samples *t*-test. Categorical data were expressed as frequencies and percentages, and between-group comparisons were performed using the chi-square (χ^2^) test. The significance level was set at α = 0.05 (two-tailed). The independent-samples *t*-test and chi-square test were chosen because the outcomes were aggregated at the day/case level and between-group comparisons were independent (different time periods without overlap). No repeated measures or clustering were present in the primary analyses.

## Results

### Comparison of baseline characteristics between the two groups

Before and after system implementation, there were no statistically significant differences in the number of actively used operating rooms, the number of nurses on duty, or the proportion of nurses assigned to subspecialty groups between the two groups (*P* > 0.05), indicating comparability. The detailed comparisons are presented in Table 1. Additional contextual variables, including surgical case mix (proportion of elective vs. emergency cases), cancellation rates, and overtime hours, were similar between the two periods based on hospital administrative data (data not shown). No major workflow changes occurred during the study period.

**TABLE 1:** Comparison of baseline characteristics between the two groups.

Variable	Control group (Jan–Jun 2025)	Experimental group (Jul–Dec 2025)	t/χ^2^	*P*
Number of actively used operating rooms (*n*, $\bar{\text{x}}$ ± s)	52.3 ± 2.1	52.7 ± 1.9	0.842	0.412
Number of nurses on duty (*n*, $\bar{\text{x}}$ ± s)	125.6 ± 3.2	126.1 ± 2.8	0.673	0.508
Proportion of nurses in subspecialty groups (%)	118/126 (93.7)	120/128 (93.8)	0.001	0.970

The denominators for the proportion of nurses in subspecialty groups (126 and 128) represent the total number of nurses on duty during each study period, which coincided with the number of valid questionnaire respondents.

### Comparison of scheduling time

The scheduling time in the experimental group was (2.0 ± 0.5) hours, which was significantly shorter than that in the control group (7.0 ± 1.2) hours, with a statistically significant difference (*t* = 8.32, *P* < 0.001). The 2-h duration included three components: (1) verification of automated assignments (approximately 30 min), (2) manual adjustment for complex or special cases (approximately 60 min), and (3) handling emergency surgery insertions (approximately 30 min). This breakdown indicates that IORSS reduces scheduling time beyond clerical tasks by automating routine matches while retaining human oversight for complex decisions. The results are shown in Table 2.

**TABLE 2:** Comparison of operating room scheduling time between the two groups (hours, $\bar{\text{x}}$ ± s).

Group	Number of days (days)	Scheduling time (hours)	t	*P*
Control group	182	7.0 ± 1.2	8.32	<0.01
Experimental group	184	2.0 ± 0.5		

The independent-samples *t*-test was used for comparison.

### Comparison of subspecialty matching accuracy

The subspecialty matching accuracy in the experimental group was 94.3% (37,909 vs. 40,200), which was significantly higher than that in the control group (78.6%, 30,340 vs. 38,600), with a statistically significant difference (χ^2^ = 418.6, *P* < 0.001). The results are shown in Table 3.

**TABLE 3:** Comparison of subspecialty matching accuracy between the two groups.

Group	Total surgical cases	Correctly matched cases	Matching accuracy (%)	χ^2^	*P*
Control group	38,600	30,340	78.6	418.6	<0.001
Experimental group	40,200	37,909	94.3		

The experimental group (Jul–Dec 2025) had 40,200 surgical cases, compared to 37,500 cases during the same months (Jul–Dec) of 2024, representing a 7.2% increase. To adjust for potential confounding factors (e.g., seasonal variation), we performed a logistic regression with matching accuracy as the dependent variable and group (experimental vs. control), case volume, month (categorical, to account for seasonality), and day of week as covariates. The adjusted matching accuracy remained significantly higher in the experimental group (OR = 4.82, 95% CI: 3.94–5.89, *P* < 0.001).

### Comparison of satisfaction with performance distribution fairness

The satisfaction rate with performance distribution fairness among nursing staff in the experimental group was 88.3% (113/128), which was significantly higher than that in the control group (61.5%, 78/126), with a statistically significant difference (χ^2^ = 9.87, *P* = 0.002). The results are shown in Table 4.

**TABLE 4:** Comparison of satisfaction with performance distribution fairness between the two groups.

Group	Number surveyed	Number satisfied	Satisfaction rate (%)	χ^2^	*P*
Control group	126	78	61.5	9.87	0.002
Experimental group	128	113	88.3		

### System operational feedback

During the implementation phase, informal feedback from schedulers and nurses indicated that the interface was intuitive, manual burden was reduced, color-coding for high-risk patients facilitated appropriate nurse assignment, and automated attendance summary minimized errors. Nurses perceived improved fairness due to transparent rules. This feedback was collected through unstructured observations and informal discussions during system deployment and pilot testing.

## Discussion

In this study, we successfully developed and implemented an Intelligent Operating Room Scheduling System (IORSS) based on the hospital information system (HIS). The system integrates three core modules, namely operating room assignment, intelligent nurse subspecialty matching, and automated attendance management, and was constructed using agile software development methods informed by literature review, clinical needs analysis, and expert panel discussions. The clinical application results demonstrated that the system significantly reduced scheduling time, improved the accuracy of matching nursing staff to surgical demands, and enhanced the perceived fairness of performance distribution. These findings not only validate the feasibility and effectiveness of the IORSS but also address the urgent need in the field to move from algorithmic validation to real-world clinical implementation (9, 10). In the following discussion, we first interpret our own findings, then relate them to the broader literature, and finally outline future directions that are not yet implemented in the current system.

Recent literature has demonstrated the potential of AI and machine learning in surgical duration prediction and scheduling optimization (7, 11–15). However, most studies remain at the algorithmic validation stage, with limited real-world implementation. In contrast to these algorithmic studies, our work provides real-world implementation evidence for a rule-based scheduling system. Three key operational improvements are worth highlighting. First, scheduling time was reduced from 7.0 ± 1.2 to 2.0 ± 0.5 h (*P* < 0.01). This reduction occurred because the rule-based system automated routine matches (surgeon-to-OR and nurse-to-subspecialty) that previously required manual lookup and negotiation. Second, subspecialty matching accuracy improved from 78.6% to 94.3% (*P* < 0.01), attributable to the structured mapping between 12 nurse subspecialty groups and surgical departments. Third, satisfaction with performance fairness increased from 61.5% to 88.3% (*P* < 0.05), likely due to the transparent, rule-based linkage between attendance, workload, and performance scores. These findings suggest that even a rule-based approach, without complex AI models, can yield substantial operational gains in resource-constrained clinical settings.

At the optimization methodology level, reinforcement learning (RL) has brought significant progress to surgical scheduling in recent years. Liu et al. (16) modeled the intraday surgical scheduling problem as a multi-agent cooperative Markov game, in which each operating room served as an agent making collaborative decisions through a centralized training with decentralized execution framework, achieving significantly superior performance over six heuristic benchmark algorithms in a simulated environment. Zhao et al. (5) proposed a novel hybrid framework integrating multi-level optimization with RL and column generation; validation using 3 years of real-world data showed a 15.8% reduction in average patient waiting time, a 5.4 percentage-point increase in operating room utilization, and a 26.2% decrease in scheduling disruptions under uncertainty. The integration of digital twin technology has further expanded the capabilities of intelligent scheduling. Silva-Aravena et al. (17) developed a prioritization framework for elective surgery that integrates patient-specific digital twins with RL. By modeling multiple indicators in real time, including clinical risk, economic factors, behavioral variables, and social vulnerability; simulation results demonstrated a 55.1% reduction in average waiting time, a 41.9% decrease in clinical risk at the time of surgery, a 16.1% increase in operating room utilization, and significantly improved priority for socially vulnerable groups. These studies indicate that AI-driven scheduling methods have shown substantial potential at the level of algorithmic performance validation; however, deployability, interpretability, and generalizability in real-world clinical settings remain critical bottlenecks that urgently need to be addressed (11). While these RL approaches demonstrate promise in simulation environments, our study shows that a simpler rule-based system can achieve meaningful real-world improvements without complex model training or large datasets.

Fairness is emerging as an increasingly prominent ethical and operational issue in the intelligent transformation of surgical scheduling. Traditional manual scheduling models often imply unequal allocation mechanisms based on seniority, historical conventions, or even informal negotiations, which are particularly disadvantageous for residents, part-time staff, and female surgeons. Wallner et al. (4) used a hierarchical RL framework to re-optimize operating room day assignments based on 1.5 years of retrospective data from plastic surgery. Their results reduced the Gini coefficient from 10.39 to 0.22, halved the variance, increased operating room exposure days for fellows from 58 to 88 days, and achieved 100% rule compliance. Kayvanfar et al. (18) constructed a comprehensive framework from the perspectives of capacity planning and fair scheduling, using a Markov queuing system to optimize resource allocation and then introducing a flexible scheduling model that considers fair patient allocation to surgeons. The FAIR framework incorporated fairness constraints, short-term demand prediction, and SHAP interpretability into the RL decision process, achieving a 45% improvement in the fairness index in simulated environments (19). These studies collectively reveal a trend: as scheduling systems evolve from purely efficiency-oriented to multi-objective optimization that balances efficiency and fairness, how to design transparent, interpretable, and auditable decision mechanisms has become a core challenge that AI-empowered surgical scheduling must address. Our findings on improved fairness satisfaction (from 61.5% to 88.3%) are consistent with this trend, suggesting that transparent rule-based systems can enhance perceived fairness even without advanced algorithmic fairness techniques.

At the level of technology implementation and industrialization, AI-driven scheduling systems are moving from proof-of-concept to real-world deployment. After deploying the Qventus surgical growth platform, the Erlanger Health System in Tennessee, USA, identified and activated underutilized operating room time slots, achieving break-even on the surgical department investment in less than 3 months, with an estimated annualized return on investment of five times and an additional 220 surgeries per month (20). LeanTaaS’s iQueue for Surgical Clinics has built a full-process coordination platform from the surgical clinic front end to the operating room; early adopters reported a 10% increase in total cases and a 75% reduction in administrative burden (21). These cases demonstrate that commercial AI scheduling platforms can generate quantifiable operational benefits in real healthcare settings. However, the scheduling rules of these platforms often focus on improving operating room utilization, with insufficient coverage of core nursing management aspects such as subspecialty matching and performance distribution. Moreover, the platform architectures are often designed for generalizability, presenting varying degrees of barriers to adaptation to individual hospital management workflows (11, 22). Notably, our system differs from these commercial platforms by integrating nursing subspecialty matching and performance linkage, addressing a gap in existing solutions.

Comparing the above domestic and international research progress with the design and application of our system reveals several notable features. First, the scheduling rules of our system are derived from the actual management processes of tertiary hospitals in China, matching operating rooms hierarchically by prioritizing the individual surgeon, followed by the surgical team leader, and finally the surgical department. This “hierarchical matching” model aligns well with the organizational structure of surgical teams in China, endowing the system with good clinical acceptability from the design stage and avoiding the situation of “algorithmically optimal but clinically rejected.” Second, our system achieves a refined breakthrough in the dimension of nursing staff management. In contrast to existing studies that mostly focus on surgeons’ time allocation, this study divided nursing staff into 12 subspecialty groups and established an association-matching mechanism between subspecialties and surgical departments. The automatic color-coding for high-risk patients (e.g., age = 70 years) and the recommendation feature for experienced nurses reflect a patient-safety-centered management philosophy. Furthermore, the linkage design between scheduling and attendance enables the system to achieve integrated closed-loop management of “scheduling, attendance, and performance,” which is rarely reported in systems described in domestic or international literature. As Bellini et al. emphasized, the true value of AI models lies in moving from predictive insights to strategic planning and actionable tools (11); our system represents a pragmatic step in this direction. Third, as the nation’s first “Internet + Healthcare” demonstration zone, Ningxia has established foundational advantages in top-level design and resource integration for healthcare informatization. As the first Intelligent Operating Room Scheduling System to be implemented in the region, our system not only fills a regional gap but also provides replicable and scalable practical experience for other tertiary hospitals in the region, serving as a useful example for advancing healthcare informatization in western China.

This study has several limitations. First, the quasi-experimental pre-/post-implementation design without a concurrent control group limits causal inference. The observed improvements could be partly attributable to temporal trends such as staff learning effects, seasonal variations in surgical volume, or other concurrent workflow changes. Second, measurement bias may exist as scheduling time was recorded by the scheduling personnel themselves rather than by independent observers, although we used timestamp logs to minimize this. Third, this is a single-center study, and the generalizability of our findings to other institutional settings with different workflows or resource constraints requires further validation. Fourth, we did not measure direct patient-level outcomes (e.g., waiting time, cancellation rates, postoperative complications) or OR utilization metrics (e.g., turnover time, overtime hours), which would provide a more comprehensive assessment of the system’s impact. Fifth, the system’s handling of dynamic insertion of emergency surgeries requires further optimization. Currently, when an emergency surgery is inserted, the system requires scheduling personnel to manually intervene to adjust the existing schedule. To address this, a multi-agent reinforcement learning framework as proposed by Liu et al. (16) could be integrated, allowing the system to dynamically reallocate ORs and nursing staff in real time while minimizing disruptions to elective surgeries. Our future work will implement and validate such a module. Sixth, although the scheduling rules of our system closely match our hospital’s actual workflows, some degree of customization will be necessary for generalization to other hospitals. Validation of generalizability will be a key direction for subsequent research. Seventh, the predictive capability of the system needs strengthening. The current version relies on information submitted by surgeons and manual fine-tuning and has not yet integrated machine learning prediction modules. Studies by Kwong et al. (14) and Park et al. (15) have fully demonstrated that machine learning models based on electronic health record data can significantly improve the accuracy of surgical duration prediction. In addition, NLP techniques can assist in extracting richer feature information from surgical descriptions (6). Future efforts could explore embedding machine learning prediction models into the scheduling engine to achieve an upgrade from “historical-rule-based” to “real-time-prediction-based” scheduling. Eighth, the scheduling rules and subspecialty grouping in our system were tailored to the workflow of a single tertiary hospital in China. Generalizing the system to other hospitals may require reconfiguration of subspecialty categories, matching priorities, and performance weighting parameters. Future studies should assess the transferability of the IORSS across different institutional settings and report the customization effort and costs involved. Ninth, although our system incorporated a hierarchical matching model (individual surgeon → team leader → department), we did not assess surgeon satisfaction with the scheduling outcomes. This is an important limitation, as surgeon acceptance is critical for real-world adoption. Future studies should include surgeon-reported outcomes. Tenth, regarding the transparency and interpretability of algorithmic decisions, which are particularly critical in healthcare settings; our study currently relies only on the ability of scheduling personnel to manually fine-tune as a safeguard mechanism and has not yet introduced interpretability tools such as SHAP to provide visual explanations of scheduling decisions (19). This is an area worth improving in future work.

In summary, the Intelligent Operating Room Scheduling System based on the hospital information system can effectively shorten scheduling time, improve the accuracy of matching nursing staff to surgical demands, optimize the fairness of performance distribution, and significantly enhance operating room resource utilization efficiency. Future research can be deepened in the following directions: first, introducing RL and multi-agent decision technologies to improve the system’s responsiveness to dynamic changes and its global optimization capability (5, 16); second, integrating machine learning and NLP techniques to enhance the system’s predictive ability for surgical duration and resource requirements (6, 14, 15); third, building decision interpretability modules to increase the transparency and clinical acceptance of scheduling results (19); and fourth, exploring deep integration of the system with other information systems such as anesthesia management, material management, and bed management, gradually constructing a smart operating room operation management platform covering the entire perioperative process (22). As the convergence of AI technology and healthcare settings deepens, surgical scheduling will move from “passive assignment” to a new intelligent stage characterized by “active prediction, dynamic optimization, and collaborative decision-making.”

## Conclusion

In this single-center pre-/post-implementation study, the Intelligent Operating Room Scheduling System significantly reduces scheduling time, improves subspecialty matching accuracy, and enhances the fairness of performance distribution, thereby providing an effective tool for refined operating room management.

## Data Availability

The original contributions presented in this study are included in this article/supplementary material, further inquiries can be directed to the corresponding author.
